# Exploring Barriers and Facilitators to COVID-19 Vaccination Uptake Among Individuals with Mental Illness in the Australian Healthcare System: A Qualitative Study Protocol

**DOI:** 10.3390/mps9030099

**Published:** 2026-06-16

**Authors:** Soumitra Das, James Killian, Mahesh Jayaram, Naveen Thomas, Chi Jonasi

**Affiliations:** 1 Mental Health and Wellbeing Services, Western Health, Melbourne 3021, Australia; 2 Department of Psychiatry, The University of Melbourne, Melbourne 3052, Australia

**Keywords:** COVID-19, vaccination, mental illness, public health, health equity, vaccine hesitancy, barriers, facilitators, qualitative research, thematic analysis

## Abstract

Individuals living with mental illness face disproportionately higher COVID-19 morbidity and mortality than the general population. Despite their prioritisation in Australia’s national vaccination rollout, vaccination rates among this population are significantly lower than those without mental illness. No previous study has employed a qualitative research paradigm to explore the barriers and facilitators to COVID-19 vaccination among people with mental illness in the Australian context. This qualitative study will employ Braun and Clarke’s reflexive thematic analysis framework. Participants will be recruited through Western Health Mental Health and Wellbeing Services in Victoria, Australia. Semi-structured individual interviews will be conducted with approximately 17–20 participants (aged 18–65) who have a DSM-5 diagnosed mental illness and any experience (vaccinated or unvaccinated) with COVID-19 vaccination. Interviews will be audio-recorded, transcribed, and then analysed and collated into themes using NVivo software. Code saturation will guide the final sample size. This study aims to produce a rich thematic map that captures the experience of individual, illness, and system-level barriers and facilitators to COVID-19 vaccination in this cohort. Findings are anticipated to inform targeted public health interventions to improve equitable vaccine uptake and contribute to closing the mortality gap for those with mental illness. This protocol has been approved by the Western Health Low Risk Ethics Panel (ERM ID: 113351).

## 1. Introduction

### 1.1. Background

The COVID-19 pandemic has had a profound impact around the world, with particular challenges for people with mental health issues. There is clear evidence demonstrating that this population has higher mortality rates and more severe health complications when infected with SARS-CoV-2 [1,2]. This is due to a range of factors, including high rates of physical comorbidities, but also the immunological consequences of mental illness itself, its impact on healthcare engagement, and the socioeconomic disadvantage associated with mental conditions [2,3]. Recognising these heightened risks, some countries, including Australia, prioritised individuals with mental health issues in their COVID-19 vaccination rollout strategies [4,5]. Despite this, vaccination rates among those with mental illness in Australia have remained far below the population average [6,7]. In attempting to close the mortality gap for this vulnerable group, there is clearly a need for an in-depth understanding of the barriers and facilitators to vaccination.

There are individual-level, illness-level, and system-level barriers and facilitators to COVID-19 vaccination in those with mental illness [8]. Existing survey research has cited a range of factors influencing hesitancy in this group, including general mistrust, misinformation and conspiracy theories, concern about side effects, safety in mental illness, and interaction with psychotropic medication [9,10]. Lack of access and systemic discrimination have also played a role in the slower vaccine rollout among this population, with strategies often overlooking the specific needs and challenges faced by individuals with mental health issues [11]. It is also important to acknowledge the strengths of this population, who have demonstrated high vaccination acceptance in some studies despite the aforementioned challenges [10]. The facilitators to vaccination, although less developed in the available literature, have included positive social group influence and understanding of the risk of infection [10]. The only previous Australian study to explore barriers and facilitators to COVID-19 vaccination in people with mental illness was a mixed-methods study conducted in Western Australia in which 233 adults with psychosis were surveyed on their vaccination status and the motivating factors behind their acceptance or refusal [12]. This research highlighted suboptimal outreach and information provision as the primary access barriers, and fear of side effects as the primary acceptance barrier in this population [12]. However, whilst this mixed-methods study provides valuable quantitative insight into access and acceptance barriers, it did not employ in-depth qualitative methodology and therefore does not capture the nuanced lived experiences, beliefs, and contextual factors that shape vaccination decision-making in this population.

To the authors’ knowledge, and based on a systematic review currently underway by the author (PROSPERO:CRD420251243939) [13], there has been no dedicated qualitative study to systematically explore the barriers and facilitators to COVID-19 vaccination among people with mental illness in the Australian context. Qualitative research is uniquely advantaged when attempting to develop a comprehensive understanding of how vaccination, its facilitators and barriers are experienced by people in this population. It is important to recognise that those living with a mental disorder are a broad and heterogeneous group, with varying beliefs, attitudes and experiences. Rather than assuming a homogenous reality and reducing complex behaviour to a set of measured variables, rigorous qualitative enquiry takes a constructivist approach that assumes multiple lived realities, and captures the lived experiences of participants, as well as the emotions, beliefs and contextual factors related to the research question [14]. This rich and person-centred information, organised thematically, provides a means for understanding the world of the participants, and can ultimately inform targeted interventions to improve vaccine uptake in the psychiatric population. Qualitative research remains underutilised in psychiatric and public health research relative to quantitative approaches, in part due to the unfamiliarity of the methodology to many researchers [15]. Publishing a detailed study protocol aims to lower this barrier by providing a transparent and reproducible methodological framework that other researchers can adapt and build upon. This is of particular importance in qualitative research, where findings are inherently context-dependent, shaped by the specific population and setting in which the study is conducted [16]. This protocol further provides ethical guidance for interacting with this vulnerable group in a qualitative research setting.

Moving towards a more nuanced and deeper understanding of vaccination behaviour in this population is not only crucial for mitigating the immediate impacts of the pandemic, but also for preparing for future public health emergencies by ensuring that vulnerable populations are not left behind in public health strategies and interventions.

### 1.2. Aim and Objectives

This study aims to explore how individuals with mental illness are accessing services through Western Health Mental Health and Wellbeing Services (WHMHWS). In Victoria, Australia, experience and navigate the barriers and facilitators to COVID-19 vaccination. Qualitative interview-based methodology will be utilised.

The specific objectives are as follows:

1. To explore how individual, illness-related, and system-level factors act as barriers to COVID-19 vaccination among people with mental illness;
2. To explore how certain factors are experienced as facilitators and motivators to COVID-19 vaccination uptake in this population;
3. To examine how participants understand and make decisions about COVID-19 vaccination, including the beliefs and experiences that inform their attitudes;
4. To develop thematic insights that may inform targeted public health interventions to improve vaccination uptake for this cohort.

## 2. Experimental Design

### 2.1. Study Design

This study adopts a qualitative, constructivist research design using Braun and Clarke’s reflexive thematic analysis to deeply explore real-life experiences through interview-based data collection [17]. The constructivist paradigm views reality as socially constructed and recognises the existence of multiple truths based on individuals’ perceptions [18]. This approach underscores the value of subjective insights, allowing for an in-depth exploration of the complex, layered realities of participants’ experiences with COVID-19 vaccination.

Semi-structured interviews will be conducted with participants aged 18–65 attending WHMHWS. Reflexive thematic analysis was selected for its flexibility and its capacity to facilitate identification and analysis of patterns in data that reflect participants’ subjective experiences [17,19]. Data collection will be manual, emphasising a thoughtful interview process and meticulous review of responses. All data and critical information will be securely stored in an encrypted, password-protected format.

### 2.2. Study Setting

The study will be conducted at WHMHWS, recruiting individuals from community mental health clinics and inpatient psychiatric settings. Face-to-face interviews will be conducted in a private interview room at Harvester Clinic, WHMHWS.

### 2.3. Eligibility Criteria

#### 2.3.1. Inclusion Criteria

The following criteria must be met for participation in this study:

- Aged between 18 and 65 years old. Individuals below 18 are excluded due to ethical considerations and consent issues related to minors. Those above 65 are excluded in this protocol as a pragmatic safeguard, given the high prevalence of age-related cognitive decline over this threshold, and that intact cognition is an eligibility criterion for this study;
- Any gender identity;
- A confirmed diagnosis of any mental illness based on DSM-5 criteria, including but not limited to depression, anxiety disorders, schizophrenia, or bipolar disorder;
- Any form of experience with COVID-19 vaccination (vaccinated, partially vaccinated, or unvaccinated);
- Intact cognition confirmed by a Montreal Cognitive Assessment (MoCA) score of ≥26;
- Capacity to provide informed consent to participate in the study;
- English-speaking with permanent residence or citizenship of Australia.

#### 2.3.2. Exclusion Criteria

- Participants below 18 or above 65 years of age;
- Individuals who cannot access vaccination due to a specific medical contraindication;
- Persons who lack capacity to consent due to the severity of their illness;
- Individuals with a MoCA score below 26, indicative of at least mild cognitive impairment;
- Non-citizens of Australia, due to differential access to the Australian healthcare system.

### 2.4. Recruitment Strategy

As participants belong to a vulnerable group, voluntary sampling (self-selection sampling) will be employed as the primary recruitment measure, allowing participants to self-identify and engage without harm to their perceived autonomy [20]. However, elements of purposeful sampling, a common method in qualitative research, will also be utilised to promote sample diversity [21]. Throughout the recruitment phase, the number of participants from each treatment setting will be monitored, and a demographic survey will be instituted at enrolment that will monitor the participants’ cultural and linguistic diversity, gender and diagnostic profile. If the emerging sample is found to be skewed homogeneously in any category, purposeful outreach within the existing recruitment sites will be undertaken to promote a diverse sample. Recruitment will occur via a poster with a QR code (Supplementary Material 5) displayed in prominent areas at WHMHWS (interview rooms, waiting areas, café, etc.). The poster will contain engaging content and graphics demonstrating the study aims and eligibility criteria. An email template has also been developed for use when potential participants contact the research team to express interest (Supplementary Material 4).

A short demographic survey (Supplementary Material 6) covering age, gender, clinical diagnosis, and vaccination status will be completed by each participant at enrolment. No specific background questions about the participant’s vaccination experience will be asked at recruitment, to prevent preconceived biases from influencing thematic development.

#### Consent

The ability to provide informed consent is a major challenge among vulnerable populations. One of the senior research investigators will contact potential participants by phone or email to explain the consent process. Written informed consent will be obtained using the Participant Information and Consent Form (Supplementary Material 2).

Participants may choose to read the PICF themselves or request that an investigator read and explain it to them. Consent capacity will be formally assessed using the principles of understanding, reasoning, retention, and communication [22]. If there are concerns regarding cognition, the MoCA will be used to assess cognitive capacity, and any score below 26 will result in exclusion. Participants will be encouraged to ask questions and clarify any concerns. Participants will be free to withdraw from the study at any time before or during the interview, without any effect on their clinical care.

### 2.5. Sample Size

Sample size in qualitative research is determined by saturation rather than statistical power. This study will employ the code saturation approach described by Hennink et al. [23,24], defined as the point at which no new codes emerge from the incoming data. This method was selected as it provides a systematic and transparent basis for determining when sufficient data has been collected, and it is well-validated for interview-based qualitative research [23]. For primary qualitative interview studies, approximately 9–17 interviews are generally sufficient to reach saturation [23]. An initial stopping criterion of 17–20 participants will be applied, with the possibility of extension if code saturation has not been reached.

Coding will be initiated from the first interview session to allow ongoing saturation monitoring. If no significant new codes emerge past the stopping criterion, code saturation will be declared [24]. However, the sample may extend beyond 20 if code saturation has not been reached.

### 2.6. Data Collection and Analysis

#### 2.6.1. Interview Design

Semi-structured, face-to-face interviews will be conducted by the Principal Investigator at Harvester Clinic, WHMHWS. Over-recruitment will be attempted to minimise the impact of participant withdrawals. The demographic survey (Supplementary Material 6) will be completed at the commencement of each interview. Participant-reported health records will be relied upon; no electronic medical records will be accessed.

A second research team member will take field notes and manage audio recording, as disclosed during the consenting process. The Principal Investigator will document observations of participant non-verbal communication and perceived emotions during the interview. Interviews will be open-ended and free-flowing, lasting approximately 30 min, as guided by the semi-structured interview schedule (Supplementary Material 1). The process will be independent of regular clinical care and withdrawal will not affect any clinical decisions. All gathered data will be de-identified.

#### 2.6.2. Data Analysis

A reflexive thematic analysis framework, as described by Braun and Clarke, will be employed [17,19]. This is among the most widely applied frameworks for thematic analysis in the social sciences and is particularly suited to constructivist, interview-based qualitative research. The following six-step framework will guide analysis [17,25]:

- Step 1—familiarisation with data: Transcripts will be read and re-read in full by at least two researchers to achieve immersion in the data. Initial observations and ideas will be noted to inform future coding.
- Step 2—generating initial codes: Data segments relevant to the research questions will be systematically coded by at least two team members. Coding will be inductive, with codes generated from the meaning within the data. Coding will be manual, utilising NVivo software (version 15) as a data management tool.
- Step 3—searching for themes: Initial codes will be collated and grouped into candidate themes by each researcher, with the aim of capturing broad patterns of shared meaning. A preliminary thematic map, utilising the mind mapping function in NVivo, will be produced to visualise relationships between themes.
- Step 4—reviewing themes: Candidate themes will be reviewed against the coded dataset and the full dataset. First, they will be reviewed against the codes to assess whether there are meaningful and coherent patterns. Following this, the themes will then be reviewed against the whole dataset to ensure they are an accurate representation. This is an iterative process in which some themes will be refined, while others will be merged or discarded. Each researcher will actively reflect on how their own assumptions and attitudes are guiding their theme generation.
- Step 5—defining and naming themes: Final themes and sub-themes will be clearly defined and named. This will occur through analytical discussion between the researchers who have been engaged in the analytic process.
- Step 6—writing up: The final themes will be reported in a format in which relevant participant responses for each theme are utilised to demonstrate the analytical process and the relationship between the data and the interpretations.

NVivo software (version 15) will be used to organise data and assist with the analysis. At least two researchers will independently code all transcripts. Coding will begin from the first interview. NVivo’s word-frequency function may be used as a supplementary aid to support initial data familiarisation; it is important to note, however, that word-frequency outputs cannot generate interpretive meaning and do not constitute analysis within a reflexive thematic analysis framework [17]. The identification, development and naming of themes will arise from the research team’s joint active reflexive engagement with the data. Analytical discussion between researchers will be used to review and refine the emerging themes. The mind mapping function in NVivo can provide a supportive role in the thematic generation process. This process is expected to produce several appropriately named themes that capture both the shared and contrasting experiences of the participants.

Reflexivity will be actively maintained throughout. Researchers will reflect on and record their personal beliefs about vaccination and experiences with individuals with severe mental illness, and consider how these may influence the research. Journaling, peer support, and external validation will be used throughout the analysis process [17].

## 3. Procedure

### 3.1. Recruitment and Screening

Recruitment posters (Supplementary Material 5) will be displayed in participant-facing areas at WHMHWS. Interested individuals will contact the research team via the QR code or email. One of the senior investigators will then contact the potential participant by phone or email to explain the study and initiate the consenting process.

Consent capacity and eligibility criteria must be assessed for all participants before enrolment.

### 3.2. Consent and Baseline Data Collection

Written informed consent will be obtained using the PICF (Supplementary Material 2). Participants may choose to read the form independently or request that an investigator read and explain it to them. Following consent, the demographic survey (Supplementary Material 6) will be administered, covering age, gender, clinical diagnosis, and vaccination status.

### 3.3. Semi-Structured Interviewing

Face-to-face interviews will be conducted at Harvester Clinic, WHMHWS. The Principal Investigator will lead each interview using the semi-structured interview schedule (Supplementary Material 1). A second team member will take field notes and manage audio recording, as disclosed during the consenting process.

Interviews will be open-ended and free-flowing. The interviewer will document observations of participant non-verbal communication and perceived emotional responses during the session. The entire interview will be audio-recorded for later transcription. The process is conducted outside of and independently from regular clinical care, and withdrawal will not affect any clinical decisions.

### 3.4. Transcription and Coding

All interviews will be audio recorded in their entirety with a recording device. A team member present from the interview will manually transcribe each recording verbatim. This process emphasises accurate depiction of the participants’ expressions. Each transcription will be in digital format on a password-protected device. All audio recordings will be deleted within 48 h of transcription. All data will be de-identified immediately following collection. Confidentiality must be maintained throughout the process.

After each interview, the transcribed data will be independently coded by at least two researchers before the next interview is conducted. Coding will be initiated from the first interview. Code saturation will be assessed iteratively after each participant’s data is coded. A minimum of two researchers will independently code all transcripts manually using NVivo (version 15). This process will continue iteratively until code saturation is confirmed as described earlier in Section 2.5. If no new codes or themes emerge past the initial stopping criterion of 17–20 participants, code saturation will be declared.

NVivo will be used as an organisational tool; all analytical decisions will be made by the research team through reflexive engagement with the data, consistent with Braun and Clarke’s framework [17]. The word-frequency function of NVivo may be used as a supplementary aid for initial familiarisation only. Mind mapping in NVivo will support the thematic generation process. Categorical themes will be generated manually from the gathered insights. Different coders’ outputs will be compared through analytical and reflexive discussion between the team members, as previously described.

### 3.5. Participant Safety and Withdrawal

#### 3.5.1. Prevention and Pre-Screening

Proactive de-escalation strategies will be employed prior to and throughout all interviews. Thorough pre-screening and continuous symptom monitoring will be conducted to identify early signs of distress (e.g., asking participants how they are feeling about the topic of vaccination or about their current life situation). If an experienced clinician predicts any sign of distress during pre-screening, the participant will not be taken for interview at that time. Environmental modifications, including use of a quiet, low-stimulation interview room, will further aid in reducing distress.

#### 3.5.2. Risk Management and Safety

Minor risks are anticipated given the nature of human participation, particularly with a vulnerable population. All participants will have access to the 24/7 Western Health mental health service.

A suicide risk assessment protocol will be implemented (Supplementary Material 3). This includes a structured set of questions assessing the participant’s current emotional state, presence of distressing thoughts, and any risk of self-harm. A risk decision tree will guide researcher response according to low-to-high-risk categorisation. Participants identified as distressed may be offered a Resource Card, a break, or a rescheduled appointment. In cases where a participant is at acute risk of self-harm, the on-site crisis team will be engaged.

#### 3.5.3. Handling of Withdrawals

Participants may withdraw at any time by notifying any member of the research team. Upon withdrawal, no further personal information will be collected. Withdrawal will have no impact on clinical care.

### 3.6. Data Security and Handling

#### 3.6.1. Data Storage

Research project data will be retained for five years in accordance with NHMRC guidelines. Hard copies of transcripts will be stored in secure cabinets. Electronic data will be stored on WHMHWS’s designated file server and backed up via the Western Health REDCap database system. User permission controls will restrict access to authorised researchers only. Unique project reference numbers (HREC/protocol numbers) will be assigned to each file.

Audio recordings will be deleted within 48 h of transcription.

#### 3.6.2. Confidentiality and Security

Western Health data management policies comply with the Australian Code for the Responsible Conduct of Research (2018) [26], the Privacy and Other Legislation Amendment Act 2024 [27], and the National Statement on Ethical Conduct in Human Research (2023) [28]. All personal identifiers (names, addresses, dates of birth, etc.) will be removed, and data will be completely de-identified. Electronic file details will be indexed in REDCap. Files will be stored in password-protected folders on WHMHWS’s computer system, requiring both REDCap login credentials and folder password access to provide an additional layer of security.

### 3.7. Reporting Guidelines

This study will be reported in accordance with the Standards for Reporting Qualitative Research (SRQR) [29]. The SRQR checklist will be completed and submitted alongside the manuscript reporting study findings.

## 4. Expected Results

The qualitative nature of this study, leveraging Braun and Clarke’s reflexive thematic analysis framework, aims to produce a rich and layered thematic map of barriers and facilitators to COVID-19 vaccination among people with mental illness. The analytic process will move iteratively from raw interview data through to increasingly abstract interpretive levels. Initial open coding of participant accounts will generate a large set of granular codes capturing discrete ideas, experiences, and meanings expressed by participants. These initial codes will then be grouped and reorganised into broader themes that aim to capture shared patterns of meaning across participants.

The resulting thematic map is expected to convey not only what barriers and facilitators exist but also the meaning participants attach to their vaccination decisions, the reasoning, emotions, ambivalence, and contextual circumstances that shape behaviour in ways that closed survey items cannot access. The themes generated will be entirely dependent on the data gathered. Examples of potential themes may include experiences of coercion or mandate pressure, the relational dimension of vaccine conversations with clinicians, and the role of peer influence within mental health service settings. Unexpected or counterintuitive themes are also anticipated, consistent with the inductive logic of the approach.

Taken together, these findings are anticipated to contribute a qualitatively grounded, contextually situated evidence base that aims to inform targeted public health interventions to improve vaccine equity for people living with mental illness in Australia.

## Data Availability

Data supporting the findings of this study will be available from the corresponding author upon reasonable request. Due to privacy and ethical restrictions associated with a vulnerable participant group, raw data will not be publicly archived.

## Supplementary Materials

The following supporting information can be downloaded at: https://www.mdpi.com/article/10.3390/mps9030099/s1, The following supporting materials were all developed specifically for the research, and are available as appendices: Supplementary Material 1—semi-structured interview schedule (V1, 10.09.2024); Supplementary Material 2—participant information and Consent Form (PICF) (V1, 10.09.2024); Supplementary Material 3—adverse event management protocol (V1, 10.09.2024); Supplementary Material 4—recruitment flowchart (V1, 10.09.2024); Supplementary Material 5—poster with QR code (V1, 10.09.2024); Supplementary Material 6—demographic survey (V1, 10.09.2024).

## Abbreviations

The following abbreviations are used in this manuscript:

DSM-5	Diagnostic and Statistical Manual of Mental Disorders, Fifth Edition
ERM ID	Ethics Reference Management Identification Number
HREC	Human Research Ethics Committee
MoCA	Montreal Cognitive Assessment
NHMRC	National Health and Medical Research Council
NVivo	Qualitative data analysis software (QSR International)
PICF	Participant Information and Consent Form
REDCap	Research Electronic Data Capture
WHMHWS	Western Health Mental Health and Wellbeing Services
