# Supplementary material for: Exploring Barriers and Facilitators to COVID-19 Vaccination Uptake Among Individuals with Mental Illness in the Australian Healthcare System: A Qualitative Study Protocol
Source: Methods Protoc. 2026 Jun 16;9(3):99. doi: 10.3390/mps9030099 (PMC13305169; doi:10.3390/mps9030099)
Supplement: Supplementary file 1 [file mps-09-00099-s001.zip › Supplementary Material 3 – Adverse event management protocol (V1, 10.09.2024).pdf]

Western Health

Participant Distress protocol

Occasionally patients will develop high levels of distress when talking about their mental health or vaccination and the events going on in their lives. Distress can also be a natural response to talking about difficult or upsetting experiences. Research participants may be experiencing acute phases of their mental illness and may exhibit irrational, confronting or potentially dangerous behaviour.

The aim of this protocol is to assist research staff to support and manage patients who develop high levels of distress during contacts with the study , so that their levels of distress drop by the end of the contact.

The main principle underpinning this distress management protocol is that talking about symptoms may escalate distress, so to de-escalate distress levels it is important to move from talking about symptoms to talking about more peripheral (but related) issues.

1. **Remember to remain calm yourself**. It can be distressing to witness someone else's distress. It is important to develop professional skills in dealing with your own distress in these circumstances. Communicating your distress to patients will only serve to escalate their distress. Remaining calm will communicate calmness to patients and will help to deescalate their distress.

2. **Continue to maintain a rapport with patients by reassuring and normalising their experience of distress**. Actively listen and reflect back to patients. Listen and validate their feelings of distress. Patients may express a range of emotions when they are feeling distressed, including sadness, despair, guilt, and anger. Be patient and allow them to vent while validating their experience of the emotions. For example:
   - "It's not uncommon for people to feel the way you're feeling when they're dealing with XXX"
   - "You've been through a tough time"
   - "You're doing a really good job talking about something that is distressing to talk about".

3. **Talk with patients about what usually works to calm them when they get upset**. For example:
   - "I can see that talking about this has been distressing for you. Now that we have finished those questions let's just take some time to try to work out how calm you feel".
   - "What things do you usually do that helps you cope with feeling upset?"
   - "What useful things has your psychologist/doctor suggested you do when you're distressed".
   - "Let's talk about some things that you might do today to relax yourself". Patients may draw from a list of calming activities to undertake once the appointment is finished. Some

examples of calming activities might include taking a warm bath, taking the dog for a walk, watch a romantic comedy, ring a friend etc.

4. **Talk with patients about what supports they have available**. “Talking or being with friends or family is often a good way to help manage distress. Have you found this useful in the past?”
   - “Who can you talk to that can support you through this?”
   - “Would it be helpful to call someone and talk more about what it’s like for you?”
   - “You don’t have to go through this on your own, often people find it helpful to talk with a friend or if they feel they need to, a supportive professional may be helpful.”

5. **Talk patients through some basic breathing techniques**. “You seem quite stressed now. Sometimes taking a moment to slow your breathing down can help to feel calmer. Maybe it would be useful if we did a quick relaxation exercise to help you drop your stress levels. Is that OK with you? ... Breathe in and out slowly through your nose-breathe in through your nose till I count to 3, now breathe out through your nose while I count to 3. Every time you breathe out say to yourself, relax”. Repeat for a few minutes.

6. **Talk through how your GP might be useful.** “Often your doctor can be helpful. He or she can talk about what sort of options you have for your distress. For example, they can talk to you about counselling, medication, or other useful treatments”.

   If necessary, offer to facilitate this by talking to the patient’s GP on their behalf, e.g., “If you like I can have a chat to your GP and let him/her know about some of the things that are bothering you that we’ve been talking about today. Then he/she can follow up with you next time you see him/her. Some people find that makes it a bit easier, so that their doctor already has an idea of what’s going on and they don’t have to raise it on their own.”

7. **Encourage patients to reengage with psychologist / counsellor if they have one.** Where patients indicate that they are seeking or has sought professional help, attempt to facilitate re-engagement with this support. You could do this by talking about whether the help sought is/was useful, how re-contact could be made, or re-enforcing skills learned previously.

8. **Ensure that patients are calm (or that distress levels have dropped to a manageable level) when you end the call/appointment**. Remind patients of the calming activity they can undertake and praise them for their efforts to soothe themselves. For example:
   - “You did a really good job in calming yourself down”
   - “You managed your distress really well”.

9. **Understand the limits of your role** – there are boundaries to how much we can and cannot do for Link-me patients and it’s important to recognise that it is not our role to give therapy but rather to acknowledge and listen to their distress and facilitate professional help if required (typically through GP referral).

All research appointments should be conducted with the following in mind:

- The appointment may be interrupted or concluded at any time should the patient not wish to continue for any reason. Opportunities to continue an appointment at a later date may be offered if appropriate. Taking a break and offering the person a refreshment break can help.
- Occasionally, completing a questionnaire or reflecting on involvement with the project may mean that a patient would like to talk further about some of the issues raised.
- Participants are under care for severe mental illness and may be in acute or florid phases of this illness, and may exhibit complex behaviours independently of their participation in the study.
- All participants will have received a resource card that lists both physical health and mental health supports that can be referred to when guiding physical and mental health-seeking behaviour

If patients become distressed and seek advice or assistance for specific issues, you should explore possible avenues for support in relation to their individual situation. This will include:

- Listening empathically to patients' account of how they are feeling and what issues they are concerned about;
- Exploring with patients whether they know anyone they feel able to talk to and encouraging them to do so if they feel this would provide understanding and support;
- Asking patients if their GP or other health professional supportive and if they feel able to discuss any physical or emotional health issues with them. If so, encouraging them to speak with the GP / health professional; if not, suggesting they look at the list of resources provided by Link-me and contact one they feel comfortable with;
- If patients disclose abuse and/or violence, elicit whether this is the first ever disclosure or if they have told others. You should also ask what current support patients have and if they like to talk to someone further about their experiences. Ask if GP or others are supportive. If not, let patients know that there are services specifically set up to support survivors of sexual abuse and / or family violence, including 1800 RESPECT (refer patient to resource list sent via email). Mandatory reporting guidelines are available at: https://aifs.gov.au/cfca/publications/mandatory-reporting-child-abuse-and-neglect In Victoria it is mandatory for registered medical professionals to report, if there are reasonable grounds, physical injury, sexual abuses and offences for a child under the age of 16 years by another person of or over the age of 18 years.

At all times the clinical responsibility for the health of the patient remains with their General Practitioner.

Distress protocol flowchart

- Patient indicates that they are experiencing stress or emotional distress

OR

- Exhibits behaviours that suggest distress (e.g. crying, trembling, etc)

Stage 1 Response

- Offer support: "**It's ok to feel upset**"
- Assess ability to continue: "**Do you feel able to carry on?**"
  - If YES: Resume
  - If NO: Go to Stage 2

Stage 2 Response

- Stop following the appointment protocol
- Offer support and assess mental status:

"I know this is tough□you're doing really well" AND
"**Tell me what thoughts you are having** OR **Tell me what you are feeling right now**"

- Review: **Do you feel able to carry on?**
  - If YES: Resume
  - If NO: Go to Stage 3

Stage 3 Response

- Identify strategies patient has previously used with success to calm down when upset
- If necessary, talk patient through basic breathing techniques
- Refer patient to the list of resources provided via email or in hard copy
- Encourage patient to contact their GP or mental health provider

OR

offer, with patient consent, to contact GP or mental health provider on the spot (if available)

Follow Up

- Encourage patient to contact GP or Lifeline if needed
- Continue trial protocol as normal
