# Supplementary material for: Exploring Barriers and Facilitators to COVID-19 Vaccination Uptake Among Individuals with Mental Illness in the Australian Healthcare System: A Qualitative Study Protocol
Source: Methods Protoc. 2026 Jun 16;9(3):99. doi: 10.3390/mps9030099 (PMC13305169; doi:10.3390/mps9030099)
Supplement: Supplementary file 1 [file mps-09-00099-s001.zip › Supplementary Material 4– Recruitment flowchart (V1, 10.09.2024);.pdf]

Western Health

Footscray Hospital
Gordon Street
Footscray VIC 3011
Tel. +61 3 0000 0000
Fax. +61 3 0000 0000
www.westernhealth.org.au

Recruitment Strategy

Recruitment Flowchart:

Recruitment Flow Chart:

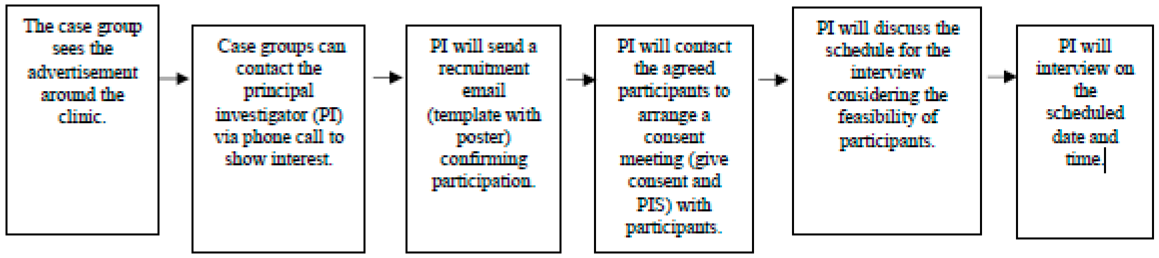

Subject Line: Mental illness and vaccination

Subhead: Your experience can change the future campaign

Dear consumers

We are clinical researchers at Western Health Mental Health and Wellbeing Services, and are working on an intended research project of "Exploring Barriers and Facilitators to COVID-19 Vaccination Uptake Among Individuals with Mental Illness in the Australian Healthcare System." This project aims to understand the facilitators and barriers faced by people with mental illnesses while getting COVID-19 vaccination coverage. We are pleased to contact you in this matter for you to consider participating in the project as your knowledge will add understanding of the risks and barriers for vulnerable groups. The benefits of your participation in the project will improve the care for the many who struggles to get vaccinated against any infectious disease.

If you are someone who has a diagnosed mental illness and experience with COVID-19 vaccination, then you match the study criteria.

You will be asked a few questions which takes around 30 minutes. There are no blood tests or scanning involved. If you want to participate, please email, or call. We will contact you and arrange a time in your clinic.

Thus, if you fit these requirements and are willing to contribute toward positive changes, your participation is highly anticipated. Thank you for your time and consideration.

Best Regards,
Dr Soumitra Das
Western Health
