# Supplementary material for: Exploring Barriers and Facilitators to COVID-19 Vaccination Uptake Among Individuals with Mental Illness in the Australian Healthcare System: A Qualitative Study Protocol
Source: Methods Protoc. 2026 Jun 16;9(3):99. doi: 10.3390/mps9030099 (PMC13305169; doi:10.3390/mps9030099)
Supplement: Supplementary file 1 [file mps-09-00099-s001.zip › Supplementary Material 1 – Semi-structured interview schedule (V1, 10.09.2024).pdf]

Form 1

Please complete the survey below.

Thank you!

1) Before proceeding, ensure participants have read and understood the participant information and consent (PICF), given consent and had the opportunity to ask questions.

Semi-structured interview questions:

Introduction:
The coverage of vaccine rollout has been significantly slower in people with mental or physical health difficulties. There is no study, which systematically explored the facilitators and barriers in vaccination roll out among people with severe mental illness in Australia. The aim of the study is "to identify the barriers to COVID 19 vaccination among people with severe mental illness. You will be asked few questions and encouraged to give your best possible answer. The questions are not about putting a view or judging you on your vaccination status. Those questions are about difficulties one might face during vaccination. I understand that you have consented for the session, and we can turn on the recorder if you are ok with that! Remind why the session is being recorded etc.

Record ON!

◯ Yes
◯ No

2) What's your thought about the COVID vaccination? Promts : Any benefits or side effects , Myths etc.

____

3) Do you have any previous experience with other vaccinations?

◯ Yes
◯ No

4) Have you received the COVID-19 vaccine? If yes, what motivated you to get vaccinated? If not, what are the reasons for your hesitancy or decision not to get vaccinated?

____

5) Have you had challenges while getting the vaccine? (If they received any of the dose)

____

6) Does you mental health has any impact on vaccination ?

____

7) Have you experienced any practical issues during the vaccination?

____

8) Has anyone like your healthcare provider or family explained about the vaccination to you? What did they say?

____

9) What were the sources of information you received information from? What do they say ?
Prompts: Social Media etc.

____

10) Closing:
What recommendations or suggestions do you have for healthcare providers, policymakers, or organizations to improve COVID-19 vaccination uptake among individuals with mental illness in Australia?

Any other point might be important regarding your thoughts around COVID or any other vaccination in general.

____
