# Supplementary material for: Exploring Barriers and Facilitators to COVID-19 Vaccination Uptake Among Individuals with Mental Illness in the Australian Healthcare System: A Qualitative Study Protocol
Source: Methods Protoc. 2026 Jun 16;9(3):99. doi: 10.3390/mps9030099 (PMC13305169; doi:10.3390/mps9030099)
Supplement: Supplementary file 1 [file mps-09-00099-s001.zip › Supplementary Material 2 – Participant Information and Consent Form (PICF) (V1, 10.09.2024).pdf]

Western Health

Western Health Low Risk Ethics Panel

Participant Information Sheet/Consent Form (PICF)

**Project Number:**	ERM ID: 113351
**Short Name of Project:**	Vaccine and mental illness
**Full Name of Project:**	Exploring Barriers and Facilitators to COVID-19 Vaccination Uptake Among Individuals with Mental Illness in the Australian Healthcare System
**Principal Researcher:**	Dr. Soumitra Das, Consultant Psychiatrist, Western Health<br>Dr Mahesh Jayaram, Western Health.
**Associate Investigators**	Dr Naveen Thomas, Western Health.<br>Chi Jonasi , Western Health
**Location:**	Western Health Mental Health and Wellbeing Services.

You are invited to take part in this research project, Exploring Barriers and Facilitators to COVID-19 Vaccination Uptake Among Individuals with Mental Illness in the Australian Healthcare System
This is because you idented yourselves to have any form of mental illness and have something to say about vaccination. The research project is aiming to identify facilitators and barriers to receive vaccination among person with mental illness.

This Participant Information Sheet/Consent Form tells you about the research project. It explains the tests and research involved. Knowing what is involved will help you decide if you want to take part in the research.

Please read this information carefully. Ask questions about anything that you don't understand or want to know more about. Before deciding whether or not to take part, you might want to talk about it with a relative, friend or local doctor.

Participation in this research is voluntary. If you don't wish to take part, you don't have to. You will receive the best possible care whether or not you take part.

If you decide you want to take part in the research project, you will be asked to sign the consent section. By signing it you are telling us that you:

- Understand what you have read.
- Consent to take part in the research project.
- Consent to the tests and research that are described.
- Consent to the use of your personal and health information as described.

You will be given a copy of this Participant Information and Consent Form to keep.

1. What is the research project about?

The COVID-19 pandemic has posed significant challenges in our lives. The mortality due to COVID-19 is significantly higher in people with physical health issues. As people with severe mental illness often suffer from multiple physical comorbidities such as Diabetes, heart disease, and obesity, the mortality risk gets multiplied. People with disabilities were being prioritized in the COVID-19 vaccination rollout by the Federal Government of Australia. However, the coverage of vaccine rollout was significantly slower in people with mental or physical health difficulties.

There are reports from 'New Zealand' which mentioned that some people with severe mental illness did not even know that they were on the priority list. Also, structural discrimination embedded into the health system often works as a barrier to access vaccination.

So far there is no study that systematically explored the barriers to vaccination rollout among people with severe mental illness in Australia. We anticipate various themes that may come up during the interview, such as lack of access, poor understanding of the need, fear of adverse reaction or interaction with existing medicine, strong belief against vaccination, and general lack of care about physical health issues. In our study, we will systematically explore the facilitators and barriers to vaccination using semi-structured questionnaires. The interview will commence by focusing on different categories of barriers such as person-related (e.g., personal choice with no specific cause, hesitancy), system-related (e.g., transport), disability-related (e.g., Poor cognitive functioning), and so on.

The aim of the study is “to identify the facilitators and barriers to COVID-19 vaccination among people with mental illness.

2. **Who is running the project?**

The project will be carried out in Western Health Mental Health and Wellbeing Services (WHMHWS). The project will include consumers of WHMHWS who suffer from any mental illnesses. Dr. Soumitra Das will be acting as the principal investigator with other Co-Investigators, all of whom are part of WHMHW services.

There are three psychiatrists and one senior nurse clinician/practitioner, one senior OT and public health researcher in the research team.

The project is funded by Otsuka Pharmaceutical as an early career research grant. The grant will be used for statistical analysis, and publication costs as per the agreement between Otsuka and WHMHWS. The researchers do not have any other relationship with Otsuka Pharmaceutical. We will not share any data with the OTSUKA or any other external agency.

Also, an important consideration is given to that participants who may feel distressed during the interview. Since the clinical research investigators (SD, MJ, NT) are all experts in managing distress from their field of expertise, especially in complex mental illnesses, they will link you with the services for safety and care at the end of the interview if necessary. The researchers will share the list of services through a resource card.

3. **Why are you asking me to take part?**

Contemporary evidence shows that people with physical or mental health illnesses have poor vaccination rollout in comparison to the general population. It is possible that a person with severe mental illness might face different barriers to getting vaccinated. We are eager to explore the barriers one might have faced in their vaccination journey. If you are experiencing mental illness and have not attained full COVID-19 vaccination status including first booster, then you are eligible for our study. If you are someone who has severe mental health issues and does not want to get vaccinated for any reason, we are also eager to know the reasons.

4. **What do I need to do in this research project?**

The research project involves interviews that will be conducted by a research investigator. Your role in the project is very important for gaining the responses during interviews. You will be asked few questions and encouraged to give your best possible answer. Those questions are about difficulties one might face during vaccination. Those questions will be asked in an individual interview session with you. You can choose the time to discuss as per your availability. You will not require any psychological or physical tests. The interview is expected to be free flowing where you can give the best and honest answer on how you feel about the vaccination. The interview does not intend to pressure you to make any decisions about vaccination, so most of the questions are likely to be open-ended.

We will record your answer using an electronic recorder. Co-Investigator may use pen and paper to write down notes on the interview during the recording process too.

One of the investigators will transcribe the recording by using statistical software like NVivo.
The interview process will take a maximum 30 minutes which will be outside the designated time for your clinical care. The interview will take place in the mental health clinic where you receive your usual care.

5. Can I withdraw from the project?

If you decide to withdraw from this research project, please notify a member of the research team before you withdraw. A member of the research team will inform you if there are any special requirements linked to withdrawing. If you do withdraw your consent during the research project, the study doctor and relevant study staff will not collect additional personal information from you, although personal information already collected will be retained to ensure that the results of the research project can be measured properly and to comply with the law. You should be aware that data collected by the sponsor up to the time you withdraw will form part of the research project results. If you do not want them to do this, you must tell them before you join the research project.

6. What are the possible benefits for me and other people in the future?

This project will help us understand the unique difficulties one with mental health issues face during vaccination. Even though the project is about COVID-19 vaccination, it can help to understand the drivers and barriers one with mental health issues might face in any other health promotion and prevention activity. Those difficulties are often not discussed in the literature. The findings might help you to reflect on your own journey in any such health prevention activity in the present moment or in the future. It can motivate you or many others with similar problems to anticipate the barriers and be prepared to overcome those with support systems.

This can help in understanding some of the myths and misinformation about vaccination too.
If you require, we can support you with educational materials. If you are ambivalent about vaccination, the interview can work as a motivational phase.

Future public health implications where the study results can be used to improve vaccination rollout among people with mental or physical disabilities.

7. What are the possible risks, side effects, and inconveniences?

As it's just an interview without any intervention, this does not have any serious side effects. However, in case you are feeling distressed with the questions and time duration, we have some effective stress-reducing strategies that are reflections of the risk management pathways. For example, we will conduct a proper risk assessment about determining how you are feeling prior to the interview to assess the possibility of you feeling distressed with questions so that we can manage the time for you. Besides, we also commit to immediately stop, give a break or postpone the interview in case you are feeling distressed at the moment as we give priority to your state of mind. These actions are also the part of resilience strategy that we aim to adopt during the interview. We will provide effective listening and empathy to every response. Also, we will provide you with a Resource card to avail of our services after the interview in case of need any services for mental health care. Such a risk management pathway can help in guiding the process of the interview effectively.
The information researchers will gather information from participants about personal histories and perspectives around COVID-19 vaccination. However, the conversations may bring up some distress among participants getting difficulties during vaccination in their previous experience. In this study, the researchers (Dr. Soumitra Das, Dr. Mahesh Jayaram, and Dr. Naveen Thomas) are experienced in managing and responding to distress in research, particularly with people with complex mental illnesses. All are trained in mental health risk and safety and are experienced, in managing any situations involving distress from participants. However, managing the different risk perceptions among study participants would require a valid risk assessment tool or method to reduce the severity of individuals facing these risks. We implied crisis services such as our 24/7 mental health services will be given to those feeling anxious and depressed after the interview as they can call back to discuss

their problems. More specifically, we have adopted a suicide risk assessment decision tree whereby an assessment would be provided to the participants to respond to certain questions for researchers to respond. For example, how they are feeling, and if they had ever intent on suicidal attempts? does your intent align with actual harming to yourself? do you think of any risk that may harm you? This assessment will help determine the low to high-risk possibilities by support from the risk decision tree to analyze the condition of patients. Not only this but the participants responding to these questions and feeling depressed can be provided with a Resource Card. Apart from this, we also adopted a Risk Management Protocol to minimize the risk of harm to patients. In the present case, for patients, we will use some strategies to reduce distress such as we can offer a break or a next appointment to complete the interview, in case if patient is feeling stressed. In more severe cases, at risk of participants self-harming themselves, the clinical researchers will call for a management team (crisis team at Harvester Clinic) who can handle the situation by de-escalating the patient. There is a separate resource card along with this form where available crisis services are mentioned.

8. How will you keep my information confidential?

Collection, use, and disclosure of a person's health information is governed by the Health Records Act 2001 (Vic) (HR Act). The Guidelines in relation to research can be obtained from the Health Services Commissioner's website: www.health.vic.gov.au/hsc.

Collection, use, and disclosure of a person's personal information is governed by the Privacy and Data Protection Act 2014 (Vic) (PDP Act). The PDP Act is administered by the Victorian Privacy Commissioner: www.privacy.vic.gov.au

The data collected or used will not be individually identifiable but re-identifiable using a code.
The data will be entered against each participant's unique Identification number.

The key to the code for re-identifiable data will be kept separately to the designated data file in a North-West Area Mental Health computer. Data will be kept in a Western Health Computer protected by a secured username and password and it can only be accessed by one of the investigators. Hard paper files will be locked in a secured Western Health cabinet for a short period such as a week after which they will be scanned and entered into the electronic database and the paper files will be shredded appropriately and as per the service policy. The computer in which the data is stored will not be taken to a premise outside the Western Health campuses.
Further actions on data management will be taken with the approval of Head of the Department and in consultation with the Western Health Office of Research and Data Trustee.

We will not access your clinical record instead we ask you about your age, gender, and diagnosis which are relevant to the project.

By signing the consent form you agree to the study team accessing health records if they are relevant to participation in this research project.

The participant's health records and any information obtained during the research project are subject to inspection (for the purpose of verifying the procedures and the data) by the relevant authorities and authorized representatives of the Sponsor, The Western Health Office of Research or as required by law. By signing the Consent Form, you authorize release of, or access to, this confidential information to the relevant study personnel and regulatory authorities as noted above.

- **Storage of information**

We may keep the research project data for 5 years as per WHMHWS research data storage policy. The research data will be stored in a secured data base run by WHMHS name redcap, also password protected shared drive and locker for hardcopies.

- **Right to access information**

You have the right to access and correct the information we collect and store. This is in line with relevant Australian and/or Victorian privacy and other relevant laws. Please contact us if you would like to access this information.

- **Publicising results**

At the end of the research project, we may present the results at conferences.

We may also publish the project results in medical journals. We will do this in a way that does not identify you.

9. How will I find out the project results?

As the result is of little relevance at individual level, we will not specifically send it to you. However, if you request, we will share the link of the publication.

10. Who should I contact for more information?

If you would like more information about the project, please contact:

Name: Dr Soumitra Das

Contact telephone: 0435095119

Email: soumitra.das@wh.org.au

In case of a medical emergency, you should call 000 or attend your nearest hospital's emergency department.

Other resources available to participants

Wilim Berrbang (Aboriginal Health Unit): Telephone: (03) 8345 0952 or email: wilim.berrbang@wh.org.au
Diversity, Equity, and Inclusion: Telephone: 0466 651 146 or email: wh-dei@wh.org.au
Disability Liaison: Telephone:0481 396 300 or email: Disabilityliaison@wh.org.au

If you have any complaints about any aspect of the project, the way it is being conducted or any questions about being a research participant in general, then you may contact the local site complaints person at Western Health.

Complaint contacts person

Position	Research Program Director, Western Health Office for Research
Telephone	(03) 8395 8073
Email	ComplaintandFeedback@wh.org.au

You will need to tell the Research Program Director/Director of Research the name of one of the researchers given in section above.

Project Number:

**Short Name of Project:**	Vaccine and mental illness
**Full Name of Project:**	Exploring Barriers and Facilitators to COVID-19 Vaccination Uptake Among Individuals with Mental Illness in the Australian Healthcare System
**Principal Researcher:**	Dr Soumitra Das , Consultant Psychiatrist, Midwest Area Mental Health Services
**Associate Investigators**	Dr Mahesh Jayaram<br>Dr Naveen Thomas

Location: **Western Health**

I have read this information statement and I understand its contents.

I understand what I have to do to be involved in this project.

I understand what risks I could face because of my involvement in this project.

I voluntarily consent to take part in this research project.

I consent to the researchers having access to my medical records in relation to the information held about my age, gender and diagnosis.

I have had an opportunity to ask questions about the project and I am satisfied with the answers I have received.

I understand that this project has been approved by the Human Research Ethics Committee. I understand that the project is required to be carried out in line with the National Statement on Ethical Conduct in Human Research (2023).

I understand I will receive a copy of this Information Statement and Consent Form.

I understand that the researcher will audio record the session.

I understand that the researcher will take notes during the session

Name of Participant (please print)
____

Signature ____________________ Date ____________________

Declaration - for participants unable to read the information and consent form
Witness to the informed consent process
Name (please print) ____________________________________

Signature ____________________ Date ____________________
* Witness is not to be the Investigator, a member of the study team or their delegate. Witness must be 18 years or older.

Declaration by the researcher: I have explained the project to the participant who has signed above. I believe that they understand the purpose, extent and possible risks of their involvement in this project.

____________________	____________________	__________
Research Team Member Name	Research Team Member Signature	Date

If you are signing this consent form you must also date your own signature
