# Supplementary material for: Exploring Barriers and Facilitators to COVID-19 Vaccination Uptake Among Individuals with Mental Illness in the Australian Healthcare System: A Qualitative Study Protocol
Source: Methods Protoc. 2026 Jun 16;9(3):99. doi: 10.3390/mps9030099 (PMC13305169; doi:10.3390/mps9030099)
Supplement: Supplementary file 1 [file mps-09-00099-s001.zip › Supplementary Material 5 – Poster with QR code (V1, 10.09.2024).pdf]

Western Health

Dept Psychiatry
Footscray Hospital
Gordon Street
Footscray VIC 3011
Tel. +61 3 0000 0000
Fax. +61 3 0000 0000
www.westernhealth.org.au

Exploring Barriers and Facilitators to COVID-19 Vaccination Uptake Among Individuals with Mental Illness in Australia Study Participation Invitation

This research study seeks participants to explore the barriers and facilitators to COVID-19 vaccination uptake among individuals with mental illness in Australia.

Inclusion Criteria:
- Age between 18 and 65 years.
- Diagnosed mental illness based on DSM-5 criteria.
- Experience with COVID-19 vaccination (received or not).
- Intact cognition and capacity to consent.
- English speaking and permanent residence or Citizenship of Australia.

Exclusion Criteria:
- Below 18 or above 65 years of age.
- Severe medical issues preventing vaccination.
- Inability to consent due to severity of illness.
- Suspected cognitive issues (MoCA score below 26).
- Non-Citizens of Australia.

This study has been reviewed and approved by Western Health Low Risk Ethics Panel.

Your participation is crucial in understanding and improving healthcare inclusivity. If you are interested in contributing to this important research, please scan the QR code below to participate.

For more information, visit our website or contact the research team, Dr Soumitra Das
Ph no. 0435095119, soumitra.das@wh.org.au

Exploring Barriers and Facilitators to COVID-19 Vaccination Uptake Among Individuals with Mental Illness in Australia

Thank you for considering participation in our study. Your involvement could help make a significant difference in the lives of many.
