# Supplementary material for: Exploring Barriers and Facilitators to COVID-19 Vaccination Uptake Among Individuals with Mental Illness in the Australian Healthcare System: A Qualitative Study Protocol
Source: Methods Protoc. 2026 Jun 16;9(3):99. doi: 10.3390/mps9030099 (PMC13305169; doi:10.3390/mps9030099)
Supplement: Supplementary file 1 [file mps-09-00099-s001.zip › Supplementary Material 6 – Demographic survey (V1, 10.09.2024).pdf]

Data Dictionary Codebook

Exploring Barriers and Facilitators to COVID-19 Vaccination Uptake Among Individuals with Mental Illness in the Australian Healthcare System (PID: 2965)

31-01-2025 16:30

**Instruments**	
Instrument	Form Name
Basic Demography Form	demographics

	#	Variable / Field Name	Field Label<br>*Field Note*	Field Attributes (Field Type, Validation, Choices, Calculations, etc.)
Instrument: **Basic Demography Form** (demographics) Enabled as survey				
	1	[record_id]	Study ID	text
	2	[introduction]	The vaccination rate for COVID-19 among individuals with mental illness is notably lower compared to the general population. Individuals with mental illness may face unique challenges that limit their access to vaccination, including fears of side effects, inadequate access, or a lack of understanding of the vaccination process. This research aims to identify both the barriers to and facilitators of COVID-19 vaccination among people with mental illness through a qualitative approach. We believe that the findings of this study will have significant public health implications, offering insights that could enhance vaccination efforts targeted at this group. It is our strong belief that people who has mental illness might be experiencing various difficulties in getting COVID vaccination. We are eager to know the themes which might be useful in future to understand the barriers and facilitators. The project will include consumers of Western Health who suffers from mental illness. The persons will be asked few questions and their answers will be recorded. The interview process will take maximum of 30 mins which is outside the designated time for clinical care. The recorded answers will be transcribed for analysis with specific software to generate different themes. The whole process with be confidential where only researchers can identify the materials. As it's interview-based research, we do not anticipate any major risk issues other than minor distress related to	dropdown<br>1 Yes<br>2 No

			questions. Are you willing to take part in the research,	
	3	[age]	Age (years)	text
	4	[sex]	Gender	radio<br>0 Female<br>1 Male<br>2 Non-binary
	5	[ethnicity]	Ethnicity	text
	6	[primary_language]	Primary Language Spoken	text
	7	[mh]	Do you have a diagnosed mental illness? If yes, please specify:	yesno<br>1 Yes<br>0 No
	8	[mh_name]	Mental illness? If yes, please specify:	text
	9	[who_diagnosed]	Who diagnosed the illness?	dropdown<br>1 GP<br>2 Psychologist<br>3 Self<br>4 Psychiatrist<br>5 Registrar<br>6 Case manager<br>7 Allied Health
	10	[covid_vaccine]	Have you received the COVID-19 vaccination	yesno<br>1 Yes<br>0 No
	11	[status]	Citizenship status	dropdown<br>1 Citizen<br>2 Not Citizen and PR
	12	[consent_contact]	Consenting to participate ?	yesno<br>1 Yes<br>0 No
	13	[contact_optional]	Contact Information	text
	14	[comments]	Comments	notes
	15	[demographics_complete]	Section Header: *Form Status*<br>Complete?	dropdown<br>0 Incomplete<br>1 Unverified<br>2 Complete
